# Predicting analysis times in randomized clinical trials with cancer immunotherapy

**DOI:** 10.1186/s12874-016-0117-3

**Published:** 2016-02-01

**Authors:** Tai-Tsang Chen

**Affiliations:** Department of Global Biometric Sciences, Bristol-Myers Squibb, Route 206 and Province Line Road, J44-05, Princeton, NJ 08540 USA; Department of Biostatistics, Columbia University, New York, NY USA

**Keywords:** Prediction of analysis time, Parametric mixture cure rate model, Cancer immunotherapy, Immuno-oncology, Ipilimumab

## Abstract

**Background:**

A new class of immuno-oncology agents has recently been shown to induce long-term survival in a proportion of treated patients. This phenomenon poses unique challenges for the prediction of analysis time in event-driven studies. If the phenomenon of long-term survival is not accounted for properly, the accuracy of the prediction based on the existing methods may be substantially compromised.

**Methods:**

Parametric mixture cure rate models with the best fit to empirical clinical trial data were proposed to predict analysis times in immuno-oncology studies during the course of the study. The proposed prediction procedure also accounts for the mechanism of action introduced by cancer immunotherapies, such as delayed and long-term survival effects.

**Results:**

The proposed methodology was retrospectively applied to a randomized phase III immuno-oncology clinical trial. Among various parametric mixture cure rate models, the Weibull cure rate model was found to be the best-fitting model for this study. The unique survival kinetics of cancer immunotherapy was captured in the longitudinal predictions of the final analysis times.

**Conclusions:**

Parametric mixture cure rate models, along with estimated long-term survival rates, probabilities of study incompletion, and expected statistical powers over time, provide immuno-oncology clinical trial researchers with a useful tool for continuous event monitoring and prediction of analysis times, such that informed decisions with quantifiable risks can be made for better resource and logistic planning.

**Electronic supplementary material:**

The online version of this article (doi:10.1186/s12874-016-0117-3) contains supplementary material, which is available to authorized users.

## Background

Due to the life-threatening nature and the unmet medical need of certain diseases, such as cancer, the majority of randomized phase III clinical trials in late-stage drug development involve interim analyses, with the possibility of early termination of the trial due to unexpectedly large treatment effects or excess toxicity. They enable clinical trial researchers to better utilize limited resources and to discontinue a regimen as soon as it has been established to have an unexpected efficacy or safety profile. At each interim analysis, an external data monitoring committee (DMC) or data safety monitoring board (DSMB) is usually charged with reviewing the accumulating data to ensure the participants enrolled in the studies are not subject to unnecessary risk.

Planning and executing analyses in event-driven studies can be challenging. First, the timing of these interim analyses is usually dependent on the number of events (i.e., information fraction), which determines the statistical stopping boundaries. Second, major operational efforts and expenses are involved to ensure that the quality of the data meets the standard of regulatory submissions, especially when the analyses could potentially lead to unblinding of the trial. Third, convening DMC members requires advanced planning. Even if a study contains only one final analysis, it is imperative that the study team closely monitors the progress of the trial. Each phase III trial rarely stands alone and usually belongs to a network of studies. A slight deviation from study assumptions established at the design stage may have a significant impact on the planning and execution of the clinical development program. Therefore, it is common practice that clinical trial researchers project the timing of interim or final analyses during the course of the study in order to make informed decisions for planning and logistical purposes.

A new class of cancer immunotherapies [1, 2] has been shown to result in a proportion of patients being non-susceptible to the event of interest, and who remain alive or disease-free even after long-term follow-up. This phenomenon is usually observed in Kaplan-Meier curves with non-zero tail probabilities. Long-term survivors have been observed in head and neck cancer [3], chronic myeloid leukemia [4–6], and advanced melanoma [7–14]. A recent pooled analysis of 1861 patients with advanced melanoma treated with ipilimumab, an anti-cytotoxic T-lymphocyte antigen-4 (CTLA-4) checkpoint inhibitor, in 10 prospective and 2 retrospective studies, showed that the overall survival (OS) curve began to plateau at 3 years with a 22 % survival rate [14]. Some patients included in this analysis had a survival follow-up of up to 10 years [14]. Patients with resected melanoma treated with interferon and pegylated interferon α-2b as adjuvant therapy also demonstrated a long-term survival rate in the range of 30 to 50 % [15–17]. In this setting, the phenomenon was also present in other time-to-event endpoints, such as recurrence-free survival or progression-free survival, and was demonstrated most recently by ipilimumab [18, 19]. Given that a subset of the patients is no longer at risk of the event of interest, such as disease recurrence or death, the study duration could be substantially longer than what is anticipated if this phenomenon is not accounted for or is misspecified at the study design stage.

Various parametric and non-parametric models have been proposed to predict the timing of analyses [20–27]. The underlying assumption of these existing methods, however, was that all patients in the study population are susceptible to the event of interest and will eventually experience an event during the monitoring window. Cure rate models have been a popular topic within the statistical literature [28–33]. However, they have not received much attention in the medical field until recently. When a long-term effect is expected, cure rate models can be a useful tool to design [34, 35] or to analyze and describe time-to-event data [36–38]. It is now recognized that clinical trial designs and analyses need to be tailored to the emerging early evidence and increasing knowledge from new therapies [35, 39]. Nevertheless, any deviation from the study assumptions may still lead to wrong estimates of study duration and statistical power, even if the novel OS attributes, such as the phenomenon of long-term effect, are built into the study design. The magnitude of the impact caused by the misspecification of the survival model in the study design has been studied elsewhere [35]. It is imperative that the study is closely monitored to ensure no unnecessary human or financial resource is wasted during conduct of the study.

Here, I propose an approach using the parametric mixture cure rate model, with the best fit to the empirical data, to predict the analysis times in immuno-oncology studies with time to event as the primary endpoint during the course of the study. In addition, the mechanism of action of cancer immunotherapies, such as a delayed or long-term survival effect, was taken into account by allowing the distribution of the expected time to event and the likelihood of the long-term effect among patients who are still at risk to be different based on their current on-study durations. Various parametric mixture cure rate models were evaluated in the context of cancer therapies with different mechanisms of action. The proposed prediction procedure was retrospectively applied to a randomized phase III clinical trial in treatment-naïve patients with advanced melanoma treated with dacarbazine with or without ipilimumab [8].

## Methods

To maintain the integrity of study conduct, treatment allocation is usually masked to the study teams in randomized clinical trials during late-stage drug development. Even if the actual treatments received by patients are known (i.e., open-label studies), the personnel directly involved in the study conduct should refrain themselves from accessing treatment records and from conducting any analysis by treatment arm during the course of the study. To mimic the real-world situation, the proposed method was performed based on the total number of events with the treatment arms combined.

### Parametric mixture cure rate model

Assume a study was conducted with *k* treatment arms. Let *N* be the total target sample size and let *N* (*τ*) denote the observed total number of randomized patients by time *τ* (relative to the first randomization) at which the prediction is performed. Similarly, let *D* (*τ*) and *G* (*τ*) represent the observed total number of events and administrative censors (i.e., patients who are still at risk at time *τ* due to data cutoff, rather than lost to follow-up). Suppose that *i*^*th*^ patients enters the study at time *E*_*i*_, where *i* = 1, 2, ⋯, *N*, and the event time *T*_*i*_ is independent of the censoring time *C*_*i*_ and the entry time *E*_*i*_. At the time of prediction *τ*, we observe the time to event or censoring *X*_*i*_ = *T*_*i*_ ∧ *C*_*i*_ ∧ (*τ* − *E*_*i*_)^+^ with censoring indicator variable of *δ*_*i*_ = *I*{*T*_*i*_ ≤ *C*_*i*_ ∧ (*τ* − *E*_*i*_)^+^}, where *a* ∧ *b* = min(*a*, *b*) and *a*^+^ = max(0, *a*).

Mixture models assume that the study population is composed of the following: individuals who will experience the event of interest (i.e., susceptible population) and those who will not (i.e., non-susceptible population). Mixture models allow simultaneous estimation of incidence and latency. Define *Y* as the indicator variable representing the susceptible population (*Y* = 0). Let *π* (***x***) be the probability of patients never experiencing the event of interest (i.e., non-susceptible population). The survival function of the entire population can be written in terms of a mixture of two independent survival distributions:

$$ s\left(t\Big|\boldsymbol{\theta},\ \boldsymbol{x},\ \boldsymbol{z}\right)=\pi \left(\boldsymbol{x}\right)+\left[1-\pi \left(\boldsymbol{x}\right)\right]s\left(t\Big|Y=0,\boldsymbol{\theta},\ \boldsymbol{z}\right), $$

where *π* (***x***) is the probability of cure with covariate vector of ***x*** = (*x*_1_, *x*_2_, ⋯, *x*_*p*_); *s*(*t*|*Y* = 0, ***θ***, ***z***) is the survival function for the susceptible population with model parameters ***θ*** and covariate vector of ***z*** = (*z*_1_, *z*_2_, ⋯, *z*_*q*_). If key prognostic factors (***x***, ***z***) were known, the prediction procedure described herein could be first applied to different subgroups of (***x***, ***z***) before pooling subgroup data sets for the overall assessment. The statistical details of the parameter estimation are presented in the supplementary material (Additional file 1). For illustration purposes, we assume the only available information at time *τ* is *X*_*i*_(*τ*) and *δ*_*i*_(*τ*).

The distribution of patients who are susceptible to the event of interest [i.e., *S*(*t*|*Y* = 0)] can take any form of the parametric or non-parametric distributions. Among the parametric models, exponential, Weibull [40], lognormal [41], and log-logistic [42] are commonly used to model time to event data. The first 3 distributions are also special cases of generalized gamma distribution. These 4 frequently used parametric distributions, as well as their counterparts in parametric mixture cure rate models along with the corresponding hazard functions are presented in the supplementary material (Additional file 2). *s*(*t*|*Y* = 0) and *s*(*t*) represent survival functions for the population that is subject to the event of interest and the entire population, respectively. Note that hazard rate *h*(*t*) is no longer constant in these models.

**Fig. 1:**
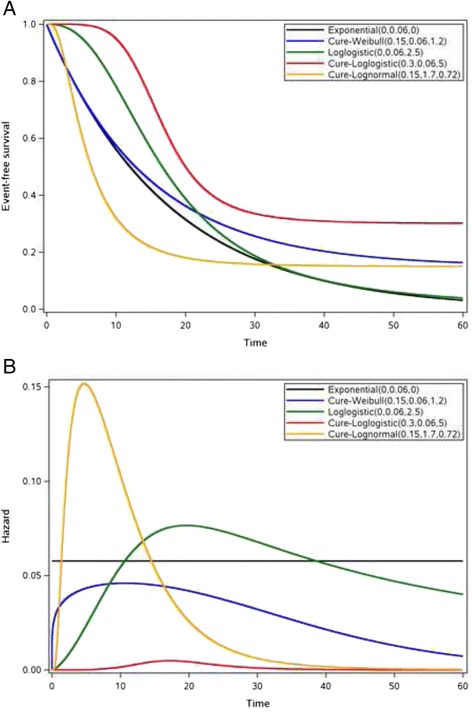
Event-free survival by mechanisms of action. **a** Treatment effects measured by time to event endpoint when patients receive therapies with different mechanisms of action (i.e., cancer immunotherapy, targeted therapy, and chemotherapy). **b** Corresponding hazard functions (i.e., risk of event of interest)

Figure 1a shows some examples of event-free survival curves based on these parametric mixture cure rate models in the context of observed or hypothesized treatment effect derived from cancer immunotherapy, targeted therapy, and cytotoxic chemotherapy in melanoma and other tumor types. The numbers associated with each model represent the parameters, defined in Additional file 2, in the order of (*π*, *θ*_1_, *θ*_2_). It has been observed that ipilimumab induces a proportion of long-term survivors and a low percentage of objective responses, although durable, resulting in long-term progression-free survival [7, 8]. The gradual decline of OS that reaches a plateau could be modeled using the Weibull cure rate model, while the lognormal cure rate model may fit the progression-free survival curve better when the data exhibited a sharp decline early on-study, followed by a plateau. The conventional exponential distribution with a constant hazard can be used to model cytotoxic chemotherapies when the survival probability is expected to drop to near zero. A log-logistic model is a viable option to model the performance of targeted therapies when early survival benefit is expected with uncertain long-term survival effect. A potential OS effect from a combination of multiple immunotherapies and/or targeted therapies can be modeled using a log-logistic cure rate model with early effect and improved long-term survival benefit. These 4 parametric mixture cure rate models can accommodate a wide range of time to event distributions, with various magnitudes of long-term benefit and different shapes of hazard function (Fig. 1b) derived from cancer therapies with different mechanisms of action.

### Prediction procedure

Due to the immaturity of accumulating data with a potential phenomenon of long-term effect during the course of the study, the shape of the survival curve is usually unstable. It is logical to fit these data with different survival distributions and select the model with the best fit to predict the timing of either interim or final analysis. The fit of the model was assessed both by graphically comparing the empirical and model-fitted curves and by using statistical criteria, such as Akaike information criterion (AIC) or Bayesian information criterion (BIC). As time elapses, the shape of the Kaplan-Meier curve may change. Therefore, different parametric mixture cure rate models may be chosen at different analysis times.

Our objective is to identify the time *T*^⋆^ at which the prespecified number of total events *D*^⋆^ for the study is reached by means of a simulation study based on the accumulating data. A proportion of *G*(*τ*) patients who represent the event-free population were randomly removed in each iteration. The remaining patients represent the population who remains at risk of the event of interest, and the future event times are obtained via the chosen survival distribution. To account for the potential mechanism of action of cancer immunotherapy, future event times are generated conditioned on the times beyond the observed times. In other words, patients who have longer on-study times at time τ will have a higher likelihood of being event-free compared with those with shorter on-study times as the time starts to level off. The point estimate and the corresponding confidence interval of the prediction are obtained from the simulation. Specifically,

1. Fit the empirical data pooled across treatment arms to the parametric mixture cure rate models and select the model with the best fit based on graphical assessment, as well as statistical criteria, such as AIC or BIC.
2. Obtain an estimate of the long-term event-free rate $\widehat{\uppi}$ and parameter estimates $\widehat{\boldsymbol{\theta}}$, with corresponding covariance matrix $\widehat{\boldsymbol{\Sigma}}$ of $\left(\widehat{\pi},\kern0.5em \widehat{\boldsymbol{\theta}}\kern0.5em \right)$.
3. If the target sample size *N* has not been reached, simulate the enrollment dates for the remaining patients.
4. Draw a random sample $\left(\tilde{\pi},\kern0.5em \tilde{\boldsymbol{\theta}}\kern0.5em \right)$ from the multivariate normal distribution with mean parameter estimates of $\left(\widehat{\pi},\kern0.5em \widehat{\boldsymbol{\theta}}\kern0.5em \right)$ and covariance matrix $\widehat{\boldsymbol{\Sigma}}$.
5. Among patients who were still at risk at time τ, a proportion of patients were selected from the risk set to represent the cured population based on the estimated distribution of the long-term event-free rate $\tilde{\pi}N/G\left(\tau \right)$.
6. For *G*(*τ*) patients with (*τ* − *E*_*i*_)^+^, the event times were generated based on the fitted parametric model exceeding the observed censored times. The random event times for patients with simulated enrollment times from Step 3 (i.e., patients have yet to be enrolled at time *τ*) were generated using the entire estimated parametric time to event curve (i.e., exceeding time zero).
7. Calculate the calendar times with newly completed data.
8. Rank the calendar times in an ascending order and identify the time *T*^⋆^ when the targeted number of events *D*^⋆^ is reached. If the completed data set from Step 7 does not yield *D*^⋆^, set *T*^⋆^ equal infinity.

Repeat steps 3–8 a large number of times to obtain a list of simulated target calendar time *T*^⋆^. Calculate the proportion of the iterations that does not contain sufficient number of patients to reach *D*^⋆^. This proportion represents the probability that the study will not conclude in a reasonable monitoring time window and can be used to determine whether the study needs to be terminated early, even if the prespecified number of events has not been reached. A high probability of study incompletion, *P*(*SIC*), could indicate a potential immaturity of the data or misspecification of the long-term event-free probability at the time of study design. The point estimate of the time of analysis is estimated from the remaining iterations by taking the median or the average of the calendar dates, and the *α*/2 and 1 − *α*/2 quantiles represent the 100(1 − *α*)% confidence limits of the prediction.

### Application

Ipilimumab is a fully human, monoclonal antibody (immunoglobulin G subclass 1) that blocks CTLA-4 binding to its ligands, B7-1 and B7-2, on antigen-presenting cells to overcome CTLA-4–mediated T-cell suppression, thus enhancing the immune response against tumors [43]. CTLA-4 expression counteracts T-cell receptor- and CD28-mediated signals to suppress the activation of T cells. The antibody blockade of CTLA-4 results in immune potentiation, augmenting T-cell activation and proliferation that causes tumor regression, and has been previously confirmed in studies with murine models [44, 45].

The proposed method was retrospectively applied to a phase III study. Permission to access the analysis data set was obtained from Bristol-Myers Squibb Company. This multicenter, randomized, double-blind 2-arm phase III study was conducted in patients with treatment-naïve stage III or IV melanoma receiving ipilimumab plus dacarbazine versus placebo plus dacarbazine [8]. The primary endpoint of the study was OS. No interim analysis with formal stopping rules was planned; however, a DMC was instituted to review efficacy and safety data biannually and to provide independent oversight of the study conduct. The original study design called for 500 randomized patients, and a total of 416 deaths were needed to provide approximately 90 % power in order to detect a 38 % increase in median OS. The study was designed under proportional hazards assumption. At the time of study initiation, it was estimated that it would take 17 months to complete the enrollment and another 17 months of follow-up with the total study duration of approximately 3 years.

During the course of the study, 6 DMC meetings were held to monitor safety and efficacy between 2008 and 2010 at approximately 6-month intervals. The study randomized 502 patients from August 2006 to February 2008. The final analysis did not take place until March of 2011, almost 2 years after what was originally anticipated. At the time of the final analysis, the study had not reached the prespecified 416 events. During the last 2 years of blinded study monitoring, it was clear that the event rate had decreased drastically, which contributed to the prolongation of the study. The long-term survival phenomenon was confirmed upon unblinding of the study [8]. The incremental number of events demonstrated a higher risk of death during the early part of the study. Eighty percent of the final 414 events were observed during the first 3 years of the study, whereas it took 2 more years to observe the remaining events.

## Results

**Fig. 2:**
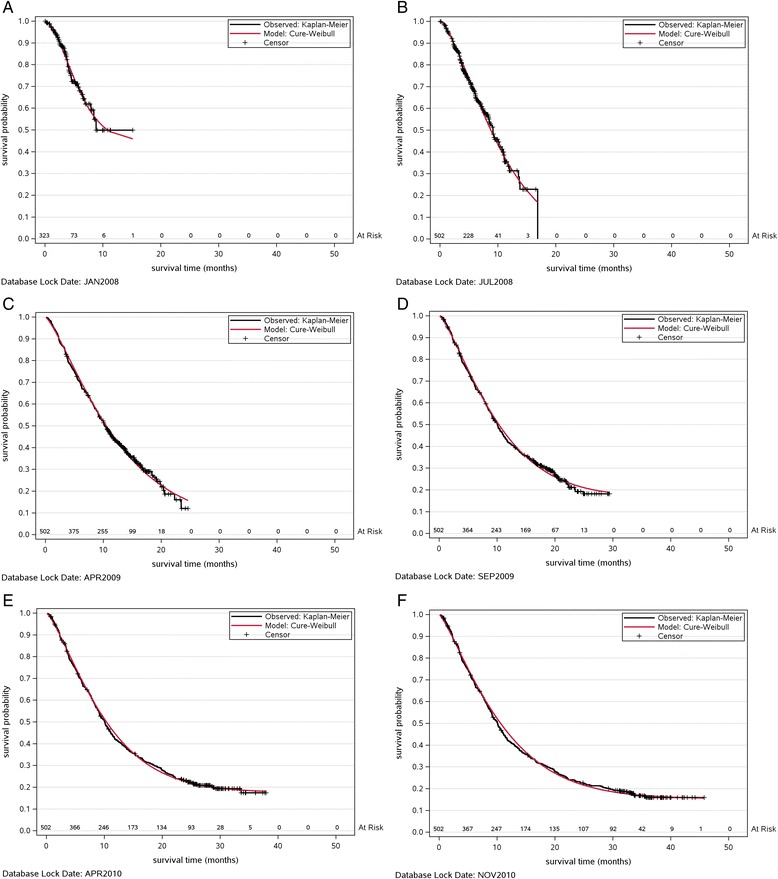
Parametric mixture cure rate modeling over time. **a**–**f** shows the observed Kaplan-Meier survival curve (*black*) and the predicted curve (*red*) fitted with Weibull mixture cure rate model using data up to prediction times between 2008 and 2010

The Weibull mixture cure rate model was the best-fitting distribution to the empirical data of this phase III randomized clinical trial, with both treatment arms combined for all 6 interim databases (Fig. 2a–f), according to AIC and BIC (Table 1).

**Table 1:** AIC and BIC from various parametric mixture cure rate models

	Goodness of Fit	CURE-EXP	CURE-WEIB	CURE-LLOG	CURE-LOGN
JAN 2008	AIC	491	367	479	484
	BIC	499	378	490	495
JUL 2008	AIC	1367	871	1337	1348
	BIC	1376	883	1350	1361
APR 2009	AIC	2461	1301	2448	2456
	BIC	2475	1314	2461	2468
SEP 2009	AIC	2695	1371	2677	2677
	BIC	2703	1383	2690	2690
APR 2010	AIC	2953	1450	2929	2932
	BIC	2961	1462	2941	2945
NOV 2010	AIC	3113	1492	3093	3093
	BIC	3121	1505	3106	3105

**Fig. 3:**
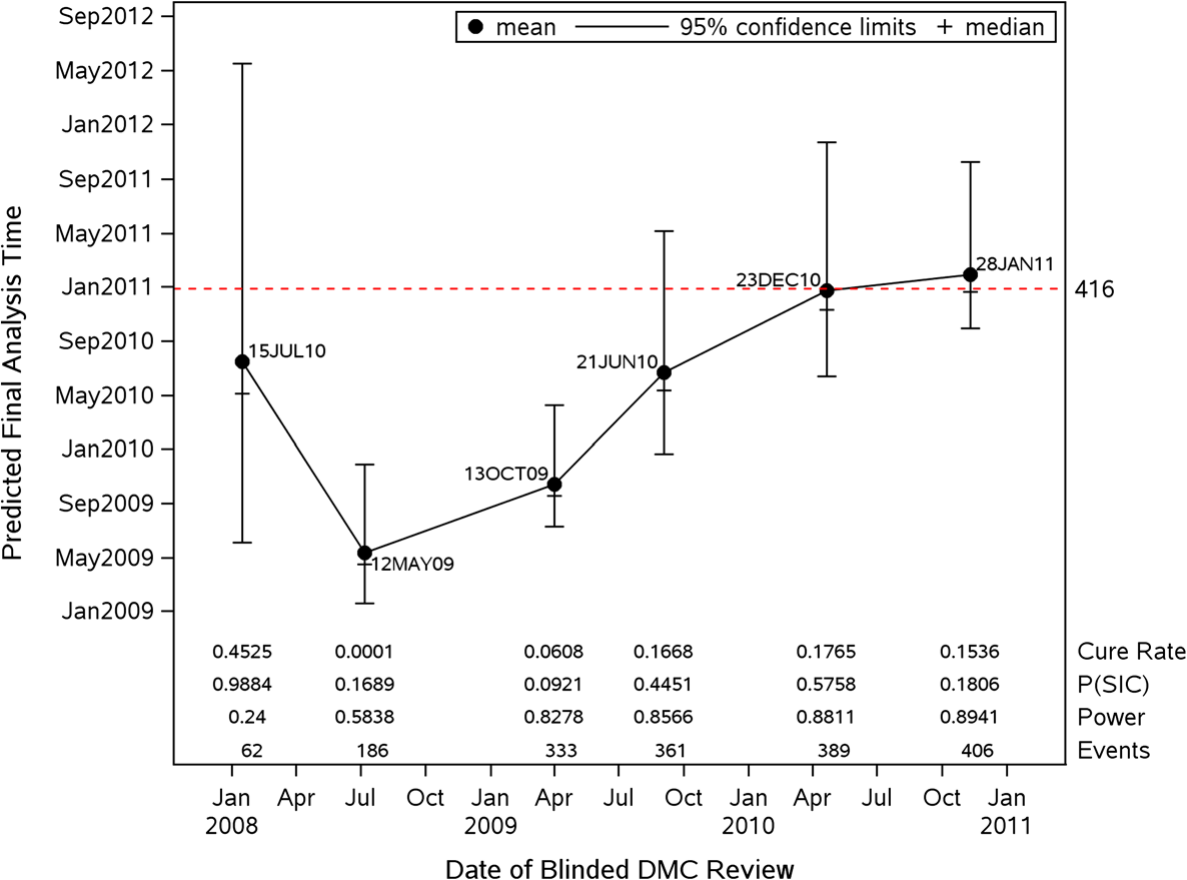
Prediction of final analysis time by parametric mixture cure rate model

Figure 3 shows the prediction of the analysis time when the prespecified final number of events (i.e., 416 events) would occur based on the Weibull mixture cure rate model. For each DMC analysis, the accumulating data were used to estimate the long-term survival rate and parameters associated with the Weibull mixture cure rate model. A simulation study with 10,000 iterations was carried out to estimate the projected calendar time of final analysis. At the time of the first DMC review in January 2008, the enrollment had not been completed. The randomization dates of these patients were simulated based on the observed enrollment rate as of the first DMC review. Because this exercise of predicting final analysis time was performed retrospectively, the actual randomization dates of those who had yet to be enrolled at the time of the first DMC meeting were available in the final database. A sensitivity analysis was conducted by taking the actual enrollment times of the patients who had yet to be enrolled at the time of first DMC review. Because the estimated and actual accrual durations were similar, i.e., 17 vs. 19 months, the sensitivity analysis did not yield notable differences. Therefore, the analysis with simulated enrollment times was presented herein. The probability of study incompletion, *P*(*SIC*), defined as the number of iterations that resulted in T* = infinity, estimated long-term survival rate from the Weibull mixture cure rate model, number of observed events, and the statistical power based on the protocol assumptions and observed number of events at each DMC review are shown at the bottom of Fig. 3.

The high study incompletion probability of 98.8 % and the relatively wide confidence interval among completed iterations in the first prediction were an indication of the immaturity of the data, at which time the enrollment had not been completed with only 62 events. The number of events tripled in the next 6 months, which led to an earlier estimate of the timing of the final analysis and lower likelihood of study incompletion (16.9 %). The estimated long-term survival rate also decreased from 45 to 0.01 % based on the accumulating data. The similar event rate continued through the third DMC analysis in April 2009. This was reflected in a narrower confidence interval of the final analysis time and relatively stable projected calendar times.

The upward trajectory of the projection and increasing width of the confidence interval for the next 2 predictions, as well as the rebounding probability of study incompletion from 9.21 to 57.6 %, indicated the decreasing event rate among remaining patients at risk. At the time of the last DMC meeting, the data showed that the probability of study incompletion dropped to 18.1 % because the observed 406 events were approaching the final number of events. The stability of the data was reflected on the estimated long-term survival rate which began to stabilize at around 16 % after the fourth DMC review in September 2009, and the associated confidence intervals of the final analysis times also covered the actual date of study completion. Although the fitted curves did not completely account for patients with the longest follow-up towards the end of the observed Kaplan-Meier curves in the second and third predictions, the subsequent three predictions with additional and more mature follow-ups confirmed the goodness-of-fit of the Cure-Weibull model. The statistical power had reached a minimum of 86 %, with more than 361 events for the last 3 DMC analyses. Nevertheless, the impact of a decreasing event rate was reflected in the delay of the analysis time projection, with the probability of study incompletion as high as 18 % when the number of events was only 10 less than the target of 416. A decision to unblind the study was made with 414 events in the first quarter of 2011.

## Discussion

Large, randomized clinical trials usually take a considerable amount of resources and time. During conduct of the trial, DSMB or DMC review is usually instituted to ensure that individuals are not exposed to unsafe or ineffective treatments. In addition, unexpected large treatment effects may also lead to early termination of the study. These activities require upfront and careful planning, especially if DMC reviews could lead to unblinding of the study. It is also not trivial to plan the timing of DMC meetings when the studies are event- driven.

A recent understanding of the biology of some diseases, such as advanced melanoma, has led to the development of a new class of cancer immunotherapies that has shown a significant survival benefit. Several immunotherapies have demonstrated delayed clinical effect and a long-term survival phenomenon, in contrast to cytotoxic chemotherapy from which patients usually derive early benefit.

Clinical trials with time-to-event endpoints are usually designed based on an exponential distribution assumption in which we assume that anything that affects the hazards does so by the same ratio at all times (i.e., proportional hazards). This implies the clinical effect of the experimental arm over the control is observed from the beginning and that the survival curves will eventually drop down to zero survival probability. The OS phenomenon exhibited by cancer immunotherapy potentially violates the constant hazard assumption, as the plateau of the survival curve implies a decreasing risk as time elapses. Therefore, it is imperative that clinical trial designs are tailored to the emerging evidence of these new therapies. Nevertheless, a slight misspecification of the study assumptions, such as the cure fraction, could still lead to a significant prolongation of the study and derail the clinical development program, even if an appropriate study design was implemented.

It has become standard practice for clinical trial researchers to predict the times of interim or final analyses during the course of the study for the purpose of resourcing and logistical planning. Various parametric or non-parametric prediction approaches have been proposed to determine the analysis times based on accumulating information of the study. These existing approaches operate under the assumption that most patients will succumb to the event of interest during the trial monitoring window.

An approach using the parametric mixture cure rate model was proposed to monitor event-driven studies when a cure fraction is anticipated. The proposed methodology was retrospectively implemented to a phase III study conducted in patients with treatment-naïve stage III or IV melanoma who received ipilimumab plus dacarbazine versus placebo plus dacarbazine. It is important to note that the use of such models should be restricted to problems in which strong biological evidence suggests the presence of such a cure fraction. Another important aspect that needs to be considered when speaking of cure is whether the duration of follow-up is sufficient. The leveling off of a Kaplan-Meier curve to a non-zero proportion, and the presence of a long and stable plateau with heavy censoring at the tail, are an indication of the presence of functional cure with reliable follow-up duration. A robust estimate of the cure fraction can only be determined with sufficiently long follow-up. The fluctuation of the cure fraction over the course of the study serves as a good indication of the maturity of the data and prevent clinical trial researchers from making untimely decisions of altering study designs. The sample size of the study should be carefully chosen to ensure the planned number of events is attainable and to allow sufficient follow-up for all patients. This has been clearly illustrated in the example provided in this article. The lack of follow-up during the first DMC analysis led to an unrealistically high and incorrect cure fraction. The stabilization of the estimated cure fraction did not occur until 1.5 years after the last patient had been randomized at the fourth DMC analysis. At the time of the final analysis, the minimum follow-up duration was close to 3 years for all patients.

It is also important to emphasize that study team personnel should refrain from accessing treatment assignments when the prediction analysis is being conducted, regardless of whether the study is blinded or open-labeled in order to maintain the study integrity. Therefore, the prediction procedure should be carried out by fitting the model to the data pooled across treatment arms. Alternatively, the proposed procedure can also be implemented by the DMC. Any decision related to drug development cannot be taken lightly, as it will have a profound impact on whether the efficacious treatments can be made available to patients in need. It is recommended that such a decision should be based on the totality of the information accumulated from the study over time, such as the probabilities of study incompletion, stabilization of the estimated long-term survival rate, and expected statistical powers. If the decision is to deviate from the original study design (e.g., unblinding the study before reaching the prespecified number of events), a dialogue with regulatory authorities is also warranted to set the expectations.

## Conclusions

A greater understanding of tumor immunology has led to the development of cancer immunotherapy. It is logical that clinical trial designs and analyses should also be tailored to the emerging early evidence and increasing knowledge about these new therapies. Parametric mixture cure rate models possess the flexibility to accommodate varying treatment effects introduced by therapies with different mechanisms of action. The proposed method provides immuno-oncology clinical trial researchers with a useful tool for continuous event monitoring and prediction of analysis times, such that informed decisions with quantifiable risks can be made for better resource and logistic planning. This will ensure that efficacious treatments are made available to patients in a timely manner.

## Additional files

Additional file 1:
**The statistical details of the parameter estimation.** (PDF 118 kb) Additional file 2:
**Commonly used conditional latency survival distribution and parametric mixture cure rate models.** (PDF 151 kb)

## Abbreviations

AIC: Akaike Information Criterion
BIC: Bayesian Information Criterion
CTLA-4: Cytotoxic T-Lymphocyte Antigen-4
DMC: Data Monitoring Committee
DSMB: Data Safety Monitoring Board
OS: overall survival

## References

[1] Melero I, Hervas-Stubbs S, Glennie M, Pardoll DM, Chen L (2007). Immunostimulatory monoclonal antibodies for cancer therapy. Nat Rev Cancer.

[2] Ott PA, Hodi S, Robert C (2013). CTLA-4 and PD-1/PD-L1 blockade: new immunotherapeutic modalities with durable clinical benefit in melanoma patients. Clin Cancer Res.

[3] Psyrri A, Kwong M, DiStasio S, Lekakis L, Kassar M, Sasaki C (2004). Cisplatin, fluorouracil, and leucovorin induction chemotherapy followed by concurrent cisplatin chemoradiotherapy for organ preservation and cure in patients with advanced head and neck cancer: long-term follow-up. J Clin Oncol.

[4] Kantarjian H, O'Brien S, Garcia-Manero G, Faderl S, Ranvandi F, Jabbour E (2012). Very long-term follow-up results of imatinib mesylate therapy in chronic phase chronic myeloid leukemia after failure of interferon alpha therapy. Cancer.

[5] Rea D, Vellenga E, Junghanß C, Baccarani M, Kantarjian H, Lofgren C (2012). Six-year follow-up of patients with imatinib-resistant or imatinib-intolerant chronic-phase chronic myeloid leukemia (CP-CML) receiving dasatinib [0199]. Haematologica.

[6] Shah NP, Kantarjian H, Kim DW, Hochhaus A, Saglio G, Guilhot F, et al. Six-year (yr) follow-up of patients (pts) with imatinib-resistant or -intolerant chronic-phase chronic myeloid leukemia (CML-CP) receiving dasatinib. J Clin Oncol. 2012;30(15s):abstract 6506.

[7] Hodi FS, O'Day SJ, McDermott DF, Weber RW, Sosman JA, Haanen JB (2010). Improved survival with ipilimumab in patients with metastatic melanoma. N Engl J Med.

[8] Robert C, Thomas L, Bondarenko I, O'Day S, Weber J, Garbe C (2011). Ipilimumab plus dacarbazine for previously untreated metastatic melanoma. N Engl J Med.

[9] Rosenberg SA. Raising the bar: the curative potential of human cancer immunotherapy. Sci Transl Med. 2012;4:127ps8. 10.1126/scitranslmed.3003634 PMC6292198 22461638

[10] Lebbé C, Weber JS, Maio M, Neyns B, Harmankaya K, Hamid O (2014). Survival follow-up and ipilimumab retreatment of patients with advanced melanoma who received ipilimumab in prior phase II studies. Ann Oncol.

[11] Maio M, Grob JJ, Aamdal S, Bondarenko I, Robert C, Thomas L (2015). Five-year survival rates for treatment-naïve patients with advanced melanoma who received ipilimumab plus dacarbazine in a phase III trial. J Clin Oncol.

[12] Robert C, Long GV, Brady B, Dutriaux C, Maio M, Mortier L (2015). Nivolumab in previously untreated melanoma without *BRAF* mutation. N Engl J Med.

[13] Robert C, Schachter J, Long GV, Arance A, Grob JJ, Mortier L (2015). Pembrolizumab versus ipilimumab in advanced melanoma. N Engl J Med.

[14] Schadendorf D, Hodi FS, Robert C, Weber JS, Margolin K, Hamid O (2015). Pooled analysis of long-term survival data from phase II and phase III trials of ipilimumab in unresectable or metastatic melanoma. J Clin Oncol.

[15] Kirkwood JM, Strawderman MH, Ernstoff MS, Smith TJ, Borden EC, Blum RH (1996). Interferon alfa-2b adjuvant therapy of high-risk resected cutaneous melanoma: the Eastern Cooperative Oncology Group Trial EST 1684. J Clin Oncol.

[16] Kirkwood JM, Ibrahim JG, Sondak VK, Richards J, Flaherty LE, Ernstoff MS (2000). High- and low-dose interferon alfa-2b in high-risk melanoma: first analysis of intergroup trial E1690/S9111/C9190. J Clin Oncol.

[17] Eggermont AM, Suciu S, Testori A, Santinami M, Kruit WH, Marden J (2012). Long-term results of the randomized phase III trial EORTC 18991 of adjuvant therapy with pegylated interferon alfa-2b versus observation in resected stage III melanoma. J Clin Oncol.

[18] Eggermont AM, Chiarion-Sileni V, Grob JJ, Dummer R, Wolchok JD, Schmidt H, et al. Ipilimumab versus placebo after complete resection of stage III melanoma: Initial efficacy and safety results from the EORTC 18071 phase III trial. J Clin Oncol. 2014;32:abstract LBA9008.

[19] Postow MA, Chesney J, Pavlick AC, Robert C, Grossmann K, McDermott D (2015). Nivolumab and ipilimumab versus ipilimumab in untreated melanoma. N Engl J Med.

[20] Rubinstein LV, Gail MH, Santner TJ (1981). Planning the duration of a comparative clinical trial with loss to follow-up and a period of continued observation. J Chronic Dis.

[21] Lachin JM, Foulkes MA (1986). Evaluation of sample size and power for analyses of survival with allowance for nonuniform patient entry, losses to follow-up, noncompliance and stratification. Biometrics.

[22] Bagiella E, Heitjan DF (2001). Predicting analysis times in randomized clinical trials. Stat Med.

[23] Ying GS, Heitjan DF, Chen TT (2004). Nonparametric prediction of event times in randomized clinical trials. Clin Trials.

[24] Donovan JM, Elliott MR, Heitjan DF (2006). Predicting event times in clinical trials when treatment arm is masked. J Biopharm Stat.

[25] Donovan JM, Elliott MR, Heitjan DF (2007). Predicting event times in clinical trials when randomization is masked and blocked. Clin Trials.

[26] Ying G, Heitjan DF (2008). Weibull prediction of event times in clinical trials. Pharm Stat.

[27] Wang J, Ke C, Jiang Q, Zhang C, Snapinn S (2011). Predicting analysis time in event-driven clinical trials with event-reporting lag. Stat Med.

[28] Boag JW (1949). Maximum likelihood estimates of the proportion of patients cured by cancer therapy. J R Stat Soc Ser B.

[29] Farewell VT (1982). The use of mixture models for the analysis of survival data with long-term survivors. Biometrics.

[30] Kuk AYC, Chen CH (1992). A mixture model combining logistic regression with proportional hazards regression. Biometrika.

[31] Chen MH, Ibrahim JG, Sinha D (1999). A new Bayesian model for survival data with a surviving fraction. J Am Stat Assoc.

[32] Sy JP, Taylor JMG (2000). Estimation in a Cox proportional hazards cure model. Biometrics.

[33] Peng Y, Dear KBG (2000). A nonparametric mixture model for cure rate estimation. Biometrics.

[34] Kim HT, Gray R (2012). Three-component cure rate model for nonproportional hazards alternative in the design of randomized clinical trials. Clin Trials.

[35] Chen TT (2013). Statistical issues and challenges in immuno-oncology. J Immunother Cancer.

[36] Chen MH, Harrington DP, Ibrahim JG (2002). Bayesian cure rate models for malignant melanoma: a case-study of Eastern Cooperative Oncology Group trial E1690. J R Stat Soc Ser C Appl Stat.

[37] Ibrahim JG, Chen MH, Chu H (2012). Bayesian methods in clinical trials: a Bayesian analysis of ECOG trials E1684 and E1690. BMC Med Res Methodol.

[38] Othus M, Barlogie B, Leblanc ML, Crowley JJ (2012). Cure models as a useful statistical tool for analyzing survival. Clin Cancer Res.

[39] Ribas A, Hersey P, Middleton MR, Gogas H, Flaherty KT, Sondak VK (2011). New challenges in endpoints for drug development in advanced melanoma. Clin Cancer Res.

[40] Carroll KJ (2003). On the use and utility of the Weibull model in the analysis of survival data. Control Clin Trials.

[41] Royston P (2001). The lognormal distribution as a model for survival time in cancer, with an emphasis on prognostic factors. Stat Neerl.

[42] Bennett S (1983). Log-logistic regression models for survival data. J R Stat Soc Series C.

[43] Hoos A, Ibrahim R, Abdallah K, Berman D, Shahabi V, Chin K (2010). Development of ipilimumab: contribution to a new paradigm for cancer immunotherapy. Semin Oncol.

[44] Leach DR, Krummel MF, Allison JP (1996). Enhancement of antitumor immunity by CTLA-4 blockade. Science.

[45] Kwon ED, Hurwitz AA, Foster BA, Madias C, Feldhaus AL, Greenberg NM (1997). Manipulation of T cell costimulatory and inhibitory signals for immunotherapy of prostate cancer. Proc Natl Acad Sci U S A.

