# Supplementary material for: Predicting analysis times in randomized clinical trials with cancer immunotherapy
Source: BMC Med Res Methodol. 2016 Feb 1;16:12. doi: 10.1186/s12874-016-0117-3 (PMC4736164; doi:10.1186/s12874-016-0117-3)
Supplement: Additional file 1: — The statistical details of the parameter estimation. (PDF 118 kb) [file 12874_2016_117_MOESM1_ESM.pdf]

Additional file 1

Since cured patients will not experience the event of interest, this leads to $\lim_{t\to\infty} S(t|Y = 1, \boldsymbol{z}) = 1$. It follows that

$$F(t|\boldsymbol{\theta}, \boldsymbol{x}, \boldsymbol{z}) = 1 - S(t|\boldsymbol{\theta}, \boldsymbol{x}, \boldsymbol{z}) = [1 - \pi(x)]F(t|Y = 0, \boldsymbol{\theta}, \boldsymbol{z})$$

The likelihood can be expressed as

$$\mathcal{L}(\boldsymbol{\theta}) = \prod_{i=1}^{N(\tau)} \{[1 - \pi_i(\boldsymbol{x})]f(t_i|Y = 0, \boldsymbol{\theta}, \boldsymbol{z})\}^{\delta_i(\tau)}\{\pi_i(\boldsymbol{x}) + [1 - \pi_i(\boldsymbol{x})]S(t_i|Y = 0, \boldsymbol{\theta}, \boldsymbol{z})\}^{1-\delta_i(\tau)},$$

and the log-likelihood is given by

$$\ell(\boldsymbol{\theta}) = \sum_{i=1}^{N(\tau)} \delta_i(\tau) \log\{[1 - \pi_i(\boldsymbol{x})]f(t_i|Y = 0, \boldsymbol{\theta}, \boldsymbol{z})\} + \sum_{i=1}^{N(\tau)} [1 - \delta_i(\tau)] \log\{\pi_i(\boldsymbol{x}) + [1 - \pi_i(\boldsymbol{x})]S(t_i|Y = 0, \boldsymbol{\theta}, \boldsymbol{z})\},$$

where $X_i(\tau)$ and $\delta_i(\tau)$ represent the observed event time and censoring status at time τ.

The maximum likelihood estimates $(\hat{\pi}, \widehat{\boldsymbol{\theta}})$ can be obtained via algorithms, such as Newton-Raphson Ridge or Trust-Region Optimization method.
