# Supplementary material for: Predicting analysis times in randomized clinical trials with cancer immunotherapy
Source: BMC Med Res Methodol. 2016 Feb 1;16:12. doi: 10.1186/s12874-016-0117-3 (PMC4736164; doi:10.1186/s12874-016-0117-3)
Supplement: Additional file 2: — Commonly used conditional latency survival distribution and parametric mixture cure rate models. (PDF 151 kb) [file 12874_2016_117_MOESM2_ESM.pdf]

**Additional file 2**

Commonly used conditional latency survival distribution and parametric mixture cure rate models

| | $S(t\|Y=0)$* | $S(t)^{\dagger}=\pi+(1-\pi)S(t\|Y=0)$ | $h(t)^{\ddagger}$ |
|---|---|---|---|
| Exponential | $e^{-\theta_1 t}$ | $\pi+(1-\pi)e^{-\theta_1 t}$ | $\frac{\theta_1(1-\pi)e^{-\theta_1 t}}{\pi+(1-\pi)e^{-\theta_1 t}}$ |
| Weibull | $e^{-(\theta_1 t)^{\theta_2}}$ | $\pi+(1-\pi)e^{-(\theta_1 t)^{\theta_2}}$ | $\frac{\theta_1\theta_2(\theta_1 t)^{\theta_2-1}(1-\pi)e^{-(\theta_1 t)^{\theta_2}}}{\pi+(1-\pi)e^{-(\theta_1 t)^{\theta_2}}}$ |
| Lognormal | $1-\Phi\left(\frac{\log(t)-\theta_1}{\theta_2}\right)$ | $1-(1-\pi)\Phi\left(\frac{\log(t)-\theta_1}{\theta_2}\right)$ | $\frac{\frac{(1-\pi)}{t\theta_2}\phi\left(\frac{\log(t)-\theta_1}{\theta_2}\right)}{1-(1-\pi)\Phi\left(\frac{\log(t)-\theta_1}{\theta_2}\right)}$ |
| Loglogistic | $\frac{1}{1+(\theta_1 t)^{\theta_2}}$ | $\pi+\frac{(1-\pi)}{1+(\theta_1 t)^{\theta_2}}$ | $\frac{(1-\pi)\theta_1\theta_2(\theta_1 t)^{\theta_2-1}}{[1+(\theta_1 t)^{\theta_2}]^2\left[\pi+\frac{(1-\pi)}{1+(\theta_1 t)^{\theta_2}}\right]}$ |

* Survival function for the population subject to event of interest;

† Survival function for the entire population;

‡ Hazard function for the entire population.
